# The provision of emergency contraception in Kinshasa's private sector pharmacies: experiences of mystery clients^☆^

**DOI:** 10.1016/j.contraception.2017.08.001

**Published:** 2018-01

**Authors:** Julie H. Hernandez, Muanda Fidèle Mbadu, Mélissa Garcia, Annie Glover

**Affiliations:** a Global Health Management and Policy, Tulane University School of Public Health and Tropical Medicine, 1440 Canal St, Suite 1900, New Orleans, LA 70112, USA; b Programme National de Santé de l'Adolescent (National Program for Adolescent Health), Ministry of Health, Avenue des Cliniques n° 43/Commune de la Gombe, Kinshasa, Democratic Republic of Congo; c International Consortium for Emergency Contraception, Management Sciences for Health, 45 Broadway #320, New York, NY 10006, USA

**Keywords:** Emergency contraception, Mystery clients, Pharmacies, Family planning, Democratic Republic of Congo

## Abstract

**Objectives:**

Recent programmatic and research efforts on addressing gaps in health systems of low-income countries increasingly see task shifting, i.e. the provision of healthcare by non-medically trained personnel, as a possible solution to increase the availability of specific services and commodities. In Kinshasa, private-sector pharmacies are the primary and preferred provider of family planning (FP) methods, and thus constitute a potential resource for expanding access to specific contraceptives. The objective of this study is to explore selected pharmacies' readiness to serve women seeking emergency contraception (EC).

**Study design:**

This study used a mystery client (MC) methodology to visit 73 pharmacies in Kinshasa, Democratic Republic of Congo (DRC). Trained interviewers posed as novice EC users and asked specific questions to evaluate the pharmacy staff's technical knowledge of EC and their attitudes towards EC clients. The results of the MC visit were recorded immediately after the MC left the pharmacy.

**Results:**

Findings indicate that more than two-thirds of EC providers were knowledgeable about EC dosage, timeframe, and side effects, and 90% were deemed helpful towards novice EC users. Rare but glaring misconceptions about EC timeframe (20% of providers) and long-term side effects (4% of providers), as well as frequent stock-out (22%) and cost issues highlight priorities for programmatic improvements.

**Conclusions:**

As new service delivery strategies are explored to complement the uneven network of health structures in DRC, this study suggests that, given proper training and integration in FP programming, private-sector pharmacies have the potential to meet specific contraceptive needs for women living in Kinshasa.

**Implication statement:**

Private pharmacies included in study sample in Kinshasa (DRC) have adequate family planning (FP) service skills to provide clients with emergency contraceptive pills.

These higher-end outlets constitute an opportunity for expanding access to FP, although, under total market approaches, a more diverse range of drugs shops should be investigated.

## 1 Introduction

The Democratic Republic of Congo (DRC), one of the 69 poorest countries in the world, is committed to the Family Planning 2020 (FP2020) global initiative to expand access to family planning (FP) information, services, and supplies, with the goal of reaching a modern contraceptive prevalence of 19.5% at the national level by 2020 [1]. As part of its engagement, the DRC has launched a number of policy, advocacy, and programmatic actions to increase the demand for modern contraceptives, reduce unmet need for FP, multiply delivery channels of FP services, and diversify the method mix available in the country.

Short-acting methods such as condoms, pills, and injectables are the most commonly used methods by women living in the capital city of Kinshasa (collectively representing 63.3% of modern method use among married and 84.3% among unmarried FP users [2]). However, among these, emergency contraception (EC) remains underused (only 1.9% of married women and 4.3% of unmarried women have ever used EC [2]). Reasons for this low EC use include limited integration in FP programs (EC only appears in official FP guidelines in cases of rape, incest, or mental incapacity [3]), very low method awareness in the population (only 22.6% of all women aged 15–49 years have heard of the method [4]), and limited distribution of the method by international partners supporting selected health facilities or pharmacies.

With 58.9% of all women aged 15–49 years reporting that their most recent birth was unintended [2] and indirect evidence of a high prevalence of unsafe abortions [5], [6]), EC's unique potential as a contraceptive “second chance” and life-saving commodity [7] represents a largely untapped resource for expanding access to FP for women with specific contraceptive needs.

Private-sector pharmacies, which are the primary source of contraceptive commodities for women in Kinshasa [2], are also the preferred service delivery points for EC in sub-Saharan countries [8]. Proximity, absence of consultation or prescription fees, and perceived discretion are key reasons why pharmacies are a promising resource to increase access to EC, including for advance provision [9]. However, pharmacy staff's lack of knowledge about EC or negative attitudes towards FP is also a potential barrier for customers. Studies conducted in India [10], Ghana [11] and Kenya [12] have shown important gaps in technical knowledge regarding EC timeframe and side effects, risk of repeat use, and additional training and support needed by pharmacists to provide reproductive health education or referrals to FP clinics [13], [14].

This study used a mystery client methodology to assess personnel's readiness in selected private pharmacies of Kinshasa to serve EC to female clients in order to explore opportunities and challenges in increasing access to EC through these types of outlets.^1^ Outcomes of interest were staff's willingness to provide EC and their knowledge of EC dosage, timeframe and side effects, obstacles to obtaining EC, and personnel's capacity to provide additional sexual and reproductive health information.

## 2 Materials and methods

To assess the conditions faced by women who try to obtain EC, we used mystery clients to visit 73 pharmacies throughout Kinshasa. This survey method uses trained people posing as real clients at a program or service delivery point and then later recording their experiences through a survey or an interview [15].

The research team recruited six mystery clients among experienced interviewers from the National Programme for Adolescent Health (PNSA) and trained them for 3 days in EC technical knowledge, mystery clients research methodology, role-playing, and practice in pharmacies near the training location. These pharmacies were not included in the study.

We then drew a sample of 73 outlets from a list of 105 pharmacies officially registered with the Ministry of Health (MoH). MoH authorization to operate requires the presence of a pharmacist with a recognized university degree as supervisor. Each of the facilities was surveyed once and did not receive advance warning of the mystery client visit.

In line with existing research on typical EC users [14], [16], mystery clients were women aged 20 to 35 years, who were trained to walk into pharmacies and request “something to avoid getting pregnant” after unprotected intercourse. During the practice role-play the mystery clients were free to develop storylines but had to include a series of questions (see Table 1) to prompt the pharmacy staff for information they may not have given spontaneously. Immediately after they exited the pharmacy, the mystery clients used a survey form to record the outcome of the visit (EC provided, refused, out of stock), questions spontaneously asked by the pharmacy staff, brand and price of EC, reasons for non-provision when relevant, and information received after asking about timeframe for use (i.e., how much time do they have to take EC before it ceases to be effective), potential side effects, and necessity of a follow-up visit. In addition, mystery clients indicated their assessment of the quality of the information they had received and their general assessment of the attitude of the pharmacy staff towards them.

**Table 1:** Questions asked about emergency contraction (EC) during mystery client visits to Kinshasa pharmacies.

1) “Hi, I have a problem. I think I′m at risk for getting pregnant and I don't want to have a child right now. Is there something I can take to avoid this?”
2) How long do I have to take this (before it stops being effective)?
3) How many pills should I take?
4) Does this pill have side-effects? Is it dangerous?
5) Should I go see a doctor later?
*[If EC is out of stock]* 6) Do you usually have it in stock? How much does it cost?
*[If EC is refused]* 7) Why can't you sell me this pill?

Table 1 details key questions mystery clients were trained to ask systematically to the pharmacy staff they interacted with.

Data collection for this research was approved by Tulane University Institutional Review Board (#16–869,451), as well as by the Ethics Committee of the Kinshasa School of Public Health (#ESP/CE/039/2016).

## 3 Results

The pharmacies visited for this study were located in 22 of the 35 health zones of Kinshasa, with an over-representation of the more modern downtown area, Gombé (18 of 73 pharmacies). In a third of the visits (*n*=25), mystery clients interacted directly with the pharmacists, while the rest of the mystery clients spoke with some other staff, such as sales person (*N*=37) or pharmacist assistant (*N*=11). One establishment included in the sample was a pharmaceutical depot specialized in product wholesale, which could provide information on EC but not the method itself.

Table 2 summarizes the main findings from mystery clients visits.

**Table 2:** Main outcomes from mystery client visits requesting Emergency contraception (EC) in Kinshasa pharmacies.

Category of information that pharmacy staff provided⁎	EC⁎⁎ obtained (*N*=54)	Percent.	EC stocked out (*N*=16)	Percent.	EC refused (*N*=3)	Percent	Total (*N*=73)	Percent.
Timeframe for use	53	98%	16	100%	-	0%	**69**	**95%**
Single dose	53	98%	**-**	0%	-	0%	**53**	**73%**
Side effects	35	65%	**-**	0%	-	0%	**35**	**48%**
Other FP methods	16	30%	4	25%	2%	1%	**22**	**30%**


Timeframe for use recommended by pharmacy staff	EC obtained (*N*=53)	Percent.	EC stocked out (*N*=16)	Percent.	EC refused (*N*=2)	Percent	Total (*N*=71)	Percent.
No more than 48 h after unprotected intercourse (incorrect)	8	15%	5	31%	1	50%	**14**	**20%**
No more than 72 h after unprotected intercourse (correct)	43	80%	11	69%	1	50%	**55**	**77%**
Read the notice	2	4%	0	0%	0	0%	**2**	**3%**
Other (incorrect)	0	0%	0	0%	0	0%	**0**	**0%**


Clients evaluation of staff attitude/respectfulness	EC obtained (*N*=54)	Percent.	EC stocked out (*N*=16)	Percent.	EC refused (*N*=3)	Percent	Total (*N*=73)	Percent.
Excellent	27	50%	3	16%	0	0%	**30**	**41%**
Correct	24	44%	10	63%	2	67%	**36**	**49%**
Mediocre	3	6%	3	16%	1	33%	**7**	**10%**

Pharmacy staff included trained pharmacists (*N*=25), assistant pharmacists (*N*=11) and shopkeepers (*N*=37).

⁎ Questions on information provided was multiple choices, hence percentages of response given may add to more than 100%.

⁎⁎ Abbreviations: EC, emergency contraception; FP, family planning; Percent., percentage.

Out of the 73 pharmacy visits, three-quarters of the clients (*n*=54) obtained EC. In 16 cases (22%), the provider was out of stock and only 3 (4%) pharmacists refused to provide the mystery clients with EC pills. Independent of the outcome of the visit (EC provided, stocked out or refused), only 11 of 73 pharmacists asked any questions when the mystery clients requested EC pills, most frequently about when unprotected intercourse had happened (*n*=10) and whether the client had ever used EC before (*n*=6). Of the 3 providers who refused to deliver EC, two insisted that a doctor's prescription was required to obtain the method, one additionally declared that he could only provide the pill to married women with the consent of their husband, and one specified that he was not comfortable with delivering the pill because it had too many side effects. In a separate case, the pharmacist explained that while she could sell the pill, her religious convictions did not allow her to provide counseling on how the method worked.

Clients who purchased EC (*n*=54) all received 1.5 mg levonorgestrel formulations. Prices ranged from 500 to 9000 CDF (US$0.50 to US$9.20) with a median of CDF 1500 (US$1.60). When asked by the mystery clients, virtually all of the 73 pharmacy staff (97%) were able to provide some information regarding the timeframe for EC use, and 53 of the 54 pharmacists who delivered EC recommended taking it in one single dose. Among the providers who shared information on timeframe (*n*=71), the majority (77%) indicated that EC has to be taken within 72 h of unprotected intercourse. 20% incorrectly said that EC should be taken immediately or less than 24 h after unprotected sex, while 3% referred the client to the insert in the package.

In addition, 65% of the pharmacy staff who provided EC (*n*=54) shared information about possible short and long-time side effects, with most of them (39 of 54) reassuring the clients that the pill had no major side effects. Three pharmacists mentioned either irregular periods or dizziness and fatigue as possible side effects. Two pharmacy staff said that women should not take EC while pregnant and another two discouraged using the method while breastfeeding. However, 12% (*n*=9) of all providers insisted that women should not use EC repeatedly, with five of them declaring that repeat or long-term use of the method could be responsible for future sterility, and in one case, uterine cancer.

Independent of the visit's results (EC provided, refused or stocked-out), 30% of the pharmacy staff offered some counseling on other FP methods, with 3 of them recommending switching to oral or injectable hormonal contraception, and 6 providing additional recommendations on safe sexual behaviors.

Finally, when completing the section of the survey form concerning the open-mindedness and general quality of service they received from the pharmacy staff, mystery clients who were refused EC were logically more likely to report a mediocre (33%) or only acceptable (67%) interaction, while mystery clients who received EC mostly declared the provider's attitude to be acceptable (44%) or excellent (50%). Independent of the visit's result however, 65 of 73 customers reported an acceptable (49%) or excellent (42%) interaction with the providers. Only two mystery clients noted distinctively hostile reactions, with the pharmacist yelling at them in one case or abruptly chasing them from their facility. In three instances, the younger mystery clients reported being asked intimate questions and lectured by the provider about abstinence, but still received EC.

## 4 Discussion

Providers' attitudes towards clients seeking EC, for the specific sample of pharmacies included in this study, did not seem to be a strong obstacle to obtaining the method. Most of the visited staff were ready to deliver the method and overall appeared helpful and non-judgmental towards the clients, two essential dimensions of service quality that are known to influence women's willingness to use health structures [17]. Stock-outs of the method was a more prevalent obstacle to accessing EC, a possible indicator of both supply chain disruption and high demand for the method, as hinted by comments from several pharmacists recorded by the mystery clients suggesting that: “*this pill is hard to find here because every time we get it in stock, women from the neighborhood come and buy it all.”* Regarding cost of the method, unpublished comparative research conducted by the International Consortium for Emergency Contraception across 72 countries using GDP data found that women in sub-Saharan Africa paid the largest percentage of their estimated weekly income for one dose of EC (23.6% in DRC). In addition, the median cost of 1500 FC for the method far exceeds household daily food budgets for the poorest segments of the population.

Moreover, providers' knowledge of EC appeared adequate to counsel clients properly on its use. While none knew that the updated World Health Organization guidelines indicate that EC, formulated as 1.5 mg levonorgestrel, is effective up to five days after unprotected intercourse [18], most of them were able to indicate a 72 h deadline consistent with the product's labeling. Nearly all pharmacy staff were aware of the one-dose take regardless of the one or two-pills packaging, and most of them provided correct counseling regarding side effects. Mystery clients also frequently reported that providers who did not immediately know the answers to questions on timeframe, dosage, or side-effects either suggested reading or did themselves read the insert inside the package.

The statistically rare but glaring misconceptions reported about EC, such as long-term risks of infertility or cancer in case of repeat use, echo perceptions and beliefs about modern FP methods frequently encountered in the DRC [20]. They raise a number of issues because pharmacists may be the only trusted source of health information in communities where access to other health structures is limited. Consequently, while pharmacists could play a pivotal role in disseminating accurate information on EC in Kinshasa, one alarming comment about the method may have a multiplier effect that could undermine method adoption. Since pharmacists are such an important source of FP methods for women in Kinshasa, these findings suggest that they should be a priority target for information and training programs.

This need for integrating pharmacists into EC programming, starting with strengthening their awareness and technical knowledge of the method, is compounded by the results of this and previous research regarding their potential bridging role towards use of regular contraceptive methods [13]. As the main source of FP commodities in the DRC, pharmacists could potentially act as a relay to introduce and refer women to other contraceptive solutions. In the present study, however, only a third of visited pharmacies provided the clients with counseling on other contraceptive methods or FP needs and solutions.

Assessing these bridging opportunities, however, would require addressing some of the present study's limitations by including a representative sample of the estimated 5000 smaller pharmacies and informal drugstores (ligablos) currently operating in Kinshasa, which are likely to be women's main source of medicines. The outlets visited for this research likely represent only the best options in terms of quality of services available. Additionally, a quarter of them were located in “downtown” Gombé, a wealthier neighborhood of Kinshasa with a high density of governmental and international organizations, which may create accessibility issues for women living in the periphery of this sprawling urban area. Further research is thus warranted to verify the extent to which the findings and opportunities suggested by this study are generalizable to a wider environment for EC provision.

In a context where total market approaches have been leveraged to increase access to a wide range of FP methods in DRC [21], the specific case of EC used in this research suggests that private pharmacies operating in Kinshasa present an opportunity to reach existing and new FP users with diverse and innovative contraceptives that could address women's specific needs and expand the method mix available in DRC. Future research needs to better define the characteristics and range of private outlets that could qualify for this type of task-shifting opportunities, as well as the policy and training efforts required to leverage their potential role as delivery points of FP services, rather than solely contraceptive providers.

## Footnotes

1 Existing guidelines from the National Programme for Reproductive Health (PNSR) in DRC indicate that EC can be accessed over the counter without prescription at private facilities, but requires a doctor's prescription for provision at public facilities. However, these guidelines are not consistent with other recommendations formulated by PNSR and it remains unclear whether they are widely known among Kinshasa's pharmacy staff.
